# Phytomanagement Reduces Metal Availability and Microbial Metal Resistance in a Metal Contaminated Soil

**DOI:** 10.3389/fmicb.2020.01899

**Published:** 2020-08-07

**Authors:** Kai Xue, Joy D. Van Nostrand, Jizhong Zhou, Silke Neu, Ingo Müller, Laura Giagnoni, Giancarlo Renella

**Affiliations:** ^1^Key Laboratory of Environmental Biotechnology, Chinese Academy of Sciences (CAS), Beijing, China; ^2^College of Resources and Environment, University of Chinese Academy of Sciences, Beijing, China; ^3^CAS Center for Excellence in Tibetan Plateau Earth Sciences, Chinese Academy of Sciences (CAS), Beijing, China; ^4^Institute for Environmental Genomics & Department of Microbiology and Plant Biology, University of Oklahoma, Norman, OK, United States; ^5^Saxon State Office for Environment, Agriculture and Geology, Freiberg, Germany; ^6^Department of Agriculture, Food, Environment and Forestry, University of Florence, Florence, Italy; ^7^Department of Agronomy, Food, Natural Resources, Animals and Environment (DAFNAE), University of Padua, Padua, Italy

**Keywords:** soil pollution, phytomanagement, soil microbial communities, microbial functional genes, soil ecological functions

## Abstract

Short rotation coppice (SRC) with metal tolerant plants may attenuate the pollution of excessive elements with potential toxicity in soils, while preserving soil resources and functionality. Here, we investigated effects of 6 years phytomanagement with willow SRC on properties including heavy metal levels, toxicity tested by BioTox, microbial biomass, enzyme activities, and functional gene abundances measured by GeoChip of soils contaminated by As, Cd, Pb and Zn, as compared to the same soils under non-managed mixed grassland representing no intervention treatment (Unt). Though metal total concentrations did not differ by SRC and Unt, SRC soils had lower metal availability and toxicity, higher organic carbon, microbial biomass, phosphatase, urease and protease activities, as compared to Unt soils. Significantly reduced abundances of genes encoding resistances to various metals and antibiotics were observed in SRC, likely attributed to reduced metal selective pressure based on less heavy metal availability and soil toxicity. SRC also significantly reduced abundances of genes involved in nitrogen, phosphorus, and sulfur cycles, possibly due to the willow induced selection. Overall, while the SRC phytomanagement did not reduce the total heavy metal concentrations in soils, it decreased the heavy metal availability and soil toxicity, which in turn led to less metal selective pressure on microbial communities. The SRC phytomanagement also reduced the abundances of nutrient cycling genes from microbial communities, possibly due to intense plant nutrient uptake that depleted soil nitrogen and phosphorus availability, and thus site-specific practices should be considered to improve the soil nutrient supply for phytomanagement plants.

## Introduction

Diffuse soil contaminations by heavy metals (HMs) and metalloids are the side effect of the worldwide industrial boom in the early to mid-1900’s, when pollution containment measures were not efficient. Panagos et al. (2013) estimated that the European Union (EU) hosts more than 8.7 × 10^5^ HM contaminated sites, of which at least 10% need urgent remediation actions as posing risks to human health. In China, the Ministry of Ecology and Environment and the Ministry of Natural Resources reported that 16% of soils exceeded the Level II requirements of the Soil Environmental Quality Standard, whereas for agricultural land, this value was as high as 19% and most of them (82%) is due to HM contaminations (Li et al., 2019).

Heavy metals accumulate in soils due to wet and dry depositions from the atmosphere and other routes, with the potential of being excessive in natural and cropped plants (Mench et al., 2010) and consequently impacting other organisms and human health through groundwater and food web (Peng et al., 2016). For example, HM contamination has been reported to reduce soil microbial biomass, diversity, and biochemical activities due to the negative selection on HM sensitive microorganisms and inhibition of microbial metabolic activities (Lejon et al., 2008; Azarbad et al., 2016). Consequently, HM contaminated soils typically contain low microbial biomass and slow SOM decomposition activity (Tyler et al., 1989), due to lower microbial biomass, functional diversity and less efficient metabolism of HM-resistant microorganisms within the soil microbial community (Mergeay, 2000).

Remediation of HM contaminated soils is generally a priority in many countries. With no intervention, HM contaminated soils undergo to revegetation by volunteer plants, but this can increase HM leaching along the soil profile due to the acidification and HM complexation with root exudates (Morel et al., 1986), increasing the risk of groundwater contamination. Though dig and dump operations or other feasible civil engineering technologies (e.g., thermal stabilization and soil washing) are main techniques currently adopted to remediate HM contaminated soils in United States, EU, and China (USEPA, 2000; EEA, 2014; Li et al., 2019), they are only applicable to relatively small areas due to high costs and difficult management of by-products. The use of civil engineering technologies also causes loss in soil fertility and ecosystem functions, which may hinder the site reuse and have long-term economic impacts on local communities (Görlach et al., 2004).

Differently, phytomanagement of HM contaminated soils is based on the use of HM stabilizing amendments and cultivation of HM tolerant plant species. Phytomanagement is increasingly adopted as an environmental securing measure to reduce risks posed by soil HM contamination to acceptable levels while preserving soil resources (Kidd et al., 2015). Among the best performing phytomanagement options, the short rotation coppice (SRC) with HM tolerant woody plants has been proven to stabilize HMs, reduce the soil toxicity and restore ecosystem functionality (Mench et al., 2010). Despite the importance of soil microbial communities as mediators for fundamental ecosystem functions, changes in functional genes encoding enzymes for carbon (C), nitrogen (N), phosphorus (P), and sulfur (S) cycles and related soil functions under phytomanagement have been addressed only by few studies (Epelde et al., 2010; Xue et al., 2015, 2018). The scarcity of data makes it still be unclear that, to what extent, microbial functional gene shifts under phytomanagement depend on types, concentration and availability of HM, and whether it is site-specific.

Here, we hypothesized that prolonged SRC management with willow could reduce HM contamination, improve soil properties and stimulate soil microbial functionality of HM contaminated soils as compared to the same soil under no intervention. We tested our hypothesis by determining abundances of microbial functional genes involved in C, N, P, and S cycling, HM and antibiotic resistances, as well as microbial biomass and enzymatic activities of soils contaminated by As, Cd, Pb, and Zn after 6 years of SRC phytomanagement willow, in comparison to the same soil under no intervention. The large genetic diversity occurs for soil microbial communities (Torsvik and Øvreås, 2002), and the impact of HM contamination on the functional genes of soil microbial communities was analyzed by the GeoChip microarray technique (Tu et al., 2014). Because the HM impact on microbial functional gene diversity and microbial activity may depend on the HM availability and soil toxicity (Kumpiene et al., 2014), we estimated HM availability by soil chemical extractions and the soil toxicity by a microbial eco-toxicological test, BioTox.

## Materials and Methods

### Site Characteristics, Management, and Sampling

The field trial was conducted at the Krummenhennersdorf site (Halsbrücke, Saxony, Germany, 50° 58′ 01.2′′ N, 13° 20′ 53.0′′ E, 300 m a.s.l.), with average annual precipitation and average annual temperature of 820 mm and 7.2°C, respectively. The soil was classified as Stagnic Luvisol (WRB, 2015). Before the beginning of the field trial, the total HM concentrations (in mg kg^–1^) were: As – 109, Cd – 3.1, Cr – 27, Cu – 24, Hg – 0.3, Ni – 18, Pb – 416, Zn – 206 (Dietzsch et al., 2011), exceeding the precautionary values of the German Federal Soil Protection Ordinance (BBodSchV, 1999) for grassland. Soil pollution in this area was due to atmospheric deposition of smelter emissions during the processing of local Pb-Zn ores. The experimental site had a surface area of 2 hectares and was set up in 2005 for demonstrating the potential of SRC with woody crops as a sustainable management for HM contaminated soils. Soils were sampled in June 2011 from five plots under *Salix viminalis cv* Tora planted in 2005, or five corresponding points of non-managed plots under mixed grassland, representing the no intervention treatment. Soils from different plots were kept separated and shipped to the analytical laboratories within 24 h after sampling. Soils were sieved with a stainless steel mesh (2 mm). Soil portions for biochemical analyses were moistened at 50% water holding capacity, soil portions used for determining chemical properties and HMs availability were air dried, whereas soils portions for the analysis of microbial functional genes were immediately stored at −80°C.

### Soil Chemical Properties, Microbial Biomass, Functional Genes, Enzyme Activities, and Toxicity

Pseudototal HMs, HM availability, total organic C (TOC), total N, inorganic N (NH_4_^+^ and NO_3_^–^), total P and available P concentration were estimated. Soil respiration rate was measured by the CO_2_ evolution, while soil microbial biomass was estimated by determining the adenosine triphosphate (ATP) content. Abundances of functional genes from soil microbial communities were analyzed by GeoChip 4.2 as reported previously (Xue et al., 2015, 2018). Activities of arylesterase, acid and alkaline phosphomonoesterase, phosphodiesterase, β-glucosidase, β-galactosidase, urease, and protease were also determined (details in Supplementary Material).

Soil toxicity was assessed by the BioTox test (Aboatox, Finland), based on the inhibition of the bioluminescence of *Vibrio fischeri* after contact with soil for 15 min, and soil was considered toxic if the bioluminescence inhibition was higher than 20% according to Lappalainen et al. (1999). BioTox test is widely adopted in the risk assessment for terrestrial ecosystems, which has been reported that the median effective concentrations (EC_50_) to *V*. *fischeri* of more than 1,200 chemical substances is comparable to responses of prokaryotes, eukaryotes and humans (Quershi et al., 1998).

### Data Analysis

All statistical analyses were performed using R version 3.0.2 (The R Foundation for Statistical Computing, Vienna, Austria), and significant differences were defined as *P* ≤ 0.05. Please see details in Supplementary Material.

## Results

### Soil Chemical Properties and Soil Toxicity

The Unt and SRC soils were with the same texture and presented similar pH values, total N and P concentrations. However, SRC soils had significantly higher TOC and significantly lower NH_4_^+^, NO_3_^–^ and available P concentrations (Table 1). Cd concentrations in SRC were close to the upper mandatory EU limits (3 mg kg^–1^) for sludge amended agricultural soils, but exceeded the level for precaution set by the German environmental legislation (BBodSchV, 1999). Compared to Unt soils, As, Cd, Pb, and Zn availability in SRC soils decreased by 39, 26, 31, and 27%, respectively (Table 2). The average bioluminescence inhibition values detected by the BioTox test in SRC (23.9% ± 2.4) were lower (*P* = 0.053) than in Unt (29.2% ± 5.2), though indicating slight ecotoxicity for both soils.

**TABLE 1:** Main physico-chemical properties in Unt and SRC soils.

**Soil management**	**pH_(H_2__O)_**	**TOC (g kg^–1^)**	***N*_tot_ (g kg^–1^)**	**C/N**	***P*_*tot*_ (g kg^–1^)**	***P*_available_ (mg kg^–1^)**	**NH_4_^+^ (mg kg^–1^)**	**NO_3_^–^ (mg kg^–1^)**
Unt	6.3	5.04	0.36	14.0	0.20	34.2	2.26	0.80
SRC	6.1	6.96**	0.47*	14.8	0.19	15.1***	1.62***	0.20***

Symbols *, **, and *** indicate significant differences between mean values in each column at *P* levels <0.05, <0.01, and <0.001, respectively.

**TABLE 2:** Total and exchangeable TE concentrations in soils under SRC or Unt management compared to German thresholds. Values in bold indicate total HM concentrations exceeding the EU thresholds for agricultural soils (CEC, 1986). Different superscripts indicate significant differences (*P* < 0.05) between mean values in each column.

**Management**	**As**	**Cd**	**Cr**	**Cu**	**Mn**	**Ni**	**Pb**	**Se**	**Zn**
**Pseudototal concentrations (mg kg^–1^)**									
Unt	**118 (±12)**	**3.1 (±0.3)**	27.7 (±2.3)	26.3 (±3.0)	842 (±45)	16.1 (±1.6)	**294 (±34)**	0.99 (±0.2)	233 (±26)
SRC	**115 (±3)**	2.9 (±0.2)	28.9 (±1.7)	23.0 (±1.4)	889 (±57)	15.9 (±1.0)	**305 (±40)**	0.92 (±0.1)	218 (±15)
Background concentrations^#1^	14	0.49	34	20	–	20	46	0.4	93
PV^#2^	20^#3^	1	60	40	–	50	70	3^#3^	150
TV^#2^	50^#4^	–	–	–	–	–	–	–	–
AV^#2^	50^#5^	20^#5^	–	200^#5,6^		1,900^#5^	1,200^#5^	–	–
**NH_4_NO_3_^–^ exchangeable concentrations (mg kg^–1^)**									
Unt	0.05 (±0.01)	0.18 (±0.02)	<0.002	0.06 (±0.01)	ND	0.02 (±0.003)	0.28 (±0.005)	ND	2.83^*a*^ (±0.25)
SRC	0.03 (±0.004)	0.17 (±0.02)	0.009 (±0.004)	0.08 (±0.002)	ND	0.03 (±0.002)	0.26 (±0.004)	ND	2.06^*b*^ (±0.19)
TV^#2^	–	–	–	–	–	–	0.1^#7^	–	–
AV^#2^	0.4^#8^	0.04^#7,9^	–	1^#8^	–	1.5^#8^	–	–	2^#8^

*^#1^90^th^ percentile of concentration in loess derived top soil under grassland (Kardel et al., 2015). ^#2^PV, precautionary values (for loamy soil); TV, trigger values, and AV, action values given by German BBodSchV (1999). ^#3^Planned precautionary value according to the draft amendment to BBodSchV (German Federal Government Act, 2017). ^#4^For arable soils if partly under anaerobic conditions, otherwise 200 mg kg^–1^. ^#5^For grassland soils to meet the EU regulation on fodder quality. ^#6^For grassland or fodder for sheep, otherwise 1,300 mg kg^–1^. ^#7^For agricultural soils to meet the EU regulation on feedstuff quality. ^#8^For agricultural soils to prevent loss of yield due to plant toxicity. ^#9^For wheat or other crops with high Cd-uptake potential, otherwise 0.1 mg kg^–1^.*

### Soil Microbial Biomass and Enzyme Activities

**FIGURE 1:**
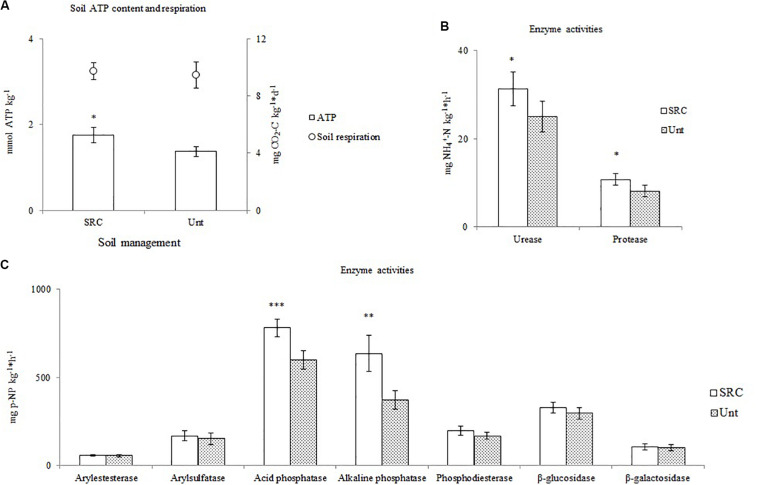
Values of soil microbial biomass (ATP content) and soil respiration **(A)**, enzyme activities involved in N cycle **(B)** and enzyme activities involved in C and P cycles **(C)** of soil under SRC or Unt management. Symbols *, ** and *** indicate significant differences between mean values *P* levels <0.05, <0.01, and 0.001, respectively.

Soils under SRC and Unt treatments showed similar values of CO_2_ evolution rate, whereas SRC soils had significantly higher (*P* < 0.05) microbial biomass than Unt soils. Unt and SRC soils showed similar values of arylesterase, arylsulfatase, phosphodiesterase, β-glucosidase and β-galactosidase activities, whereas the SRC soils had significantly higher values of acid and alkaline phosphomonoesterase, urease and protease activities (Figure 1).

### Functional Gene Abundance and Diversity

The total probe number of functional genes detected by GeoChip across all soil samples was 31,064. Among them, 29,050 and 27,384 were in Unt and SRC soils, respectively. Most (81.7%) of detected functional gene probes were present in both treatments, while 11.8 and 6.5% of detected probes were unique to Unt and SRC soils, respectively (Table 3). Richness, Shannon–Weaver (H) and Simpson Reciprocal (1/D) indices of functional gene diversity did not differ significantly between Unt and SRC soils (Table 3).

**TABLE 3:** Functional gene uniqueness and overlapping between Unt and SRC soils, and diversity indices. Values of diversity indices are presented as mean ± standard error.

**Indices**	**Treatments**		
	**Unt**	**SRC**	**Unt/SRC genetic overlap**
Unique/overlapping genes	3,680 (11.8%)	2,014 (6.5%)	25,370 (81.7%)
Gene numbers	29,050	27,384	

	**Unt**	**SRC**	***t*/*p*-value**

Richness	23,069 ± 700	21,921 ± 490	1.34/0.11
Shannon-Weaver (H)	10.0 ± 0.03	9.99 ± 0.02	1.33/0.11
InvSimpson (1/D)	23,004 ± 697	21,860 ± 488	1.34/0.11

**FIGURE 2:**
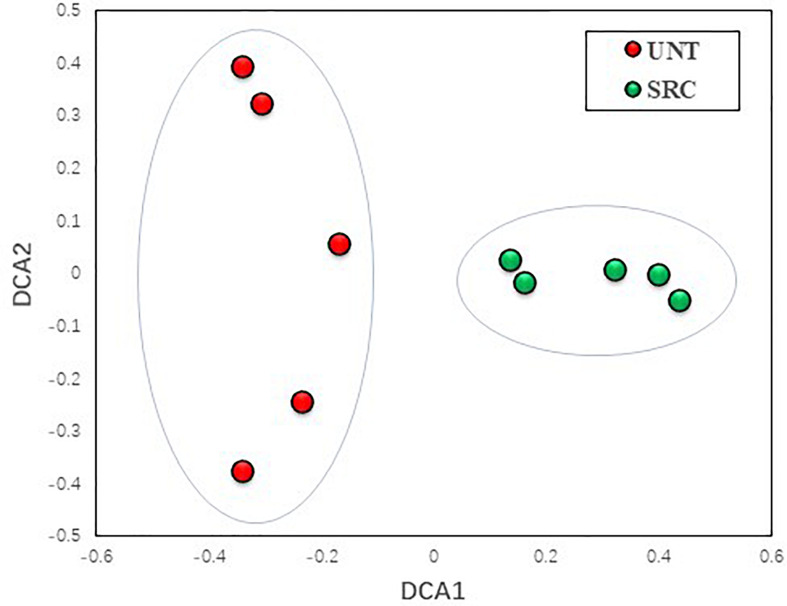
Detrended correspondence analysis of the functional gene compositions in Unt and SRC soils.

However, the DCA profile showed a clear separation between Unt and SRC soils along the DCA 1, revealing distinct microbial functional gene compositions depending on treatments (Figure 2). The MRPP, ADONIS, and ANOSIM dissimilarity tests also showed significant (*P* ≤ 0.05) differences in microbial functional gene compositions of Unt and SRC soils (Table 4). Correlation tests were performed between DCA1 and functional gene abundances to identify genes with higher discriminatory ability for treatments, and 92 of 657 genes were correlated with DCA1 significantly. Among them, 12 genes encodes enzymes for metal resistance, six genes were for stress catalog, six genes were for C degradation, four genes were for N cycling, and oner gene was for antibiotic resistance (Supplementary Table S3).

**TABLE 4:** Non-parametric analyses to test dissimilarity of communities between Unt and SRC treatments. All three tests are multivariate analyses based on *Bray*-*Curtis, Horn* and *Euclidean* dissimilarity indexes.

**SRC vs Unt**	**ADONIS^1^**		**ANOSIM^2^**		**MRPP^3^**	
	***F***	***P*^4^**	***R***	***P***	**δ**	***P***
Bray-Curtis	2.012	0.011	0.424	0.009	0.181	0.009
Horn	2.083	0.009	0.436	0.008	0.164	0.013
Euclidean	1.505	0.010	0.492	0.006	85.866	0.006

*^1^Permutational multivariate analysis of variance using distance matrices. Significance tests were performed by *F*-tests based on sequential sums of squares from permutations of the raw data. ^2^Analysis of similarities. Statistic *R* is based on the difference of mean ranks between groups and within groups. The significance of observed *R* is assessed by permuting the grouping vector to obtain the empirical distribution of *R* under null-model. ^3^Multi-response permutation procedure. Statistic δ is the overall weighted mean of within-group means of the pairwise dissimilarities among sampling units. The significance test is the fraction of permuted deltas that are less than the observed delta. ^4^*P*-value of corresponding significance test.*

Most of genes (29 of 33) encoding enzymes for degrading organic C compounds did not significantly differ in their abundance between soils under Unt and SRC, with a few exceptions (see Supplementary Material). Abundances of some functional genes involved in nutrient cycling were significantly lower in SRC than Unt soils, including *nirA*, *narG*, *nirS*, *nirK*, *amoA*, and *nifH* for N cycling, *ppx* for P cycling, *cysI, dsrB*, and *dsrA* for S cycling (see Supplementary Material). Differently, the *cysJ* encoding the sulfite reductase was significantly more abundant in SRC than Unt soils (Supplementary Figure S3).

**FIGURE 3:**
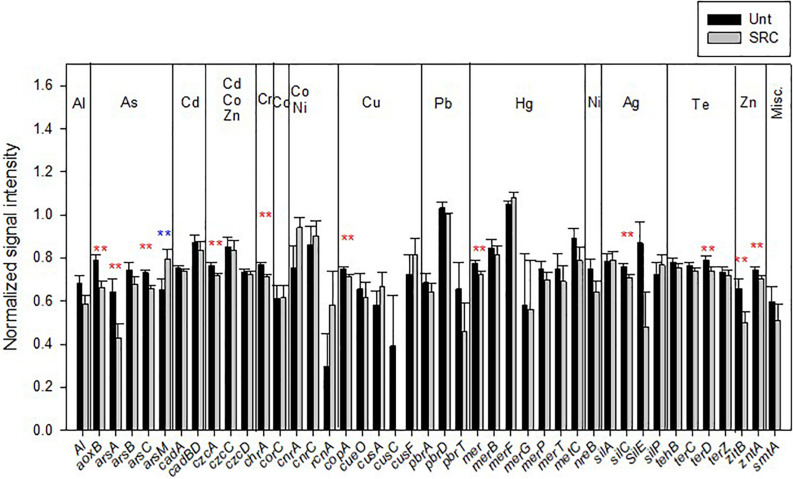
Normalized signal intensity of detected genes encoding for heavy metal(loid) resistance in the Unt and SRC soils. Error bars represent standard error. Symbols ** indicates significant differences at *P* < 0.05, with red color when SRC < Unt, with blue color when SRC > Unt.

Forty-three functional genes conferring HM resistance were detected in studied soils (Figure 3). Those encoding for As-resistance (*aoxB, arsA, and arsC*), *czcA* conferring resistance to Cd, Zn and Co, Zn resistance genes (*zntA* and *zitB*), genes conferring resistance to Cu (*copA*), Hg (*mer*), Ag (*silC)*, Te (*terD*), and Cr (*chrA*) showed significantly (*P* < 0.05) lower abundances in SRC than in Unt soils. Genes encoding for Pb resistance had lower abundance in SRC than in Unt soils, though not significantly; whereas only the As-resistance gene *arsM* was significantly (*P* < 0.05) higher in SRC than Unt soils.

**FIGURE 4:**
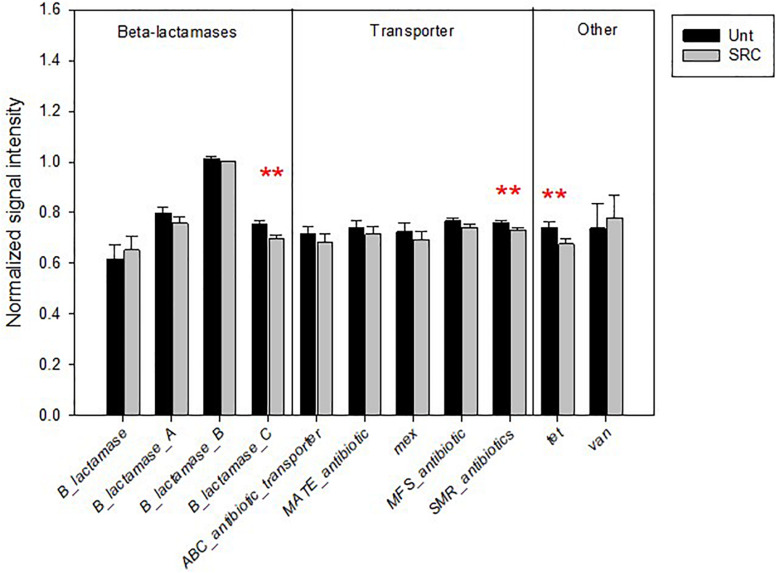
Normalized signal intensity of detected genes encoding for antibiotic resistance in the Unt and SRC soils. Error bars represent standard error. Symbols ** indicates significant differences at *P* < 0.05, with red color when SRC < Unt.

Most functional genes (nine of 11) encoding for antibiotic resistance had lower abundances in SRC than Unt soils, significantly for genes encoding for the class C of β-lactamases, small multidrug resistance (SMR) protein and *tet* for the methylcytosine dioxygenase (*P* < 0.05) (Figure 4).

### Relationship Between Microbial Functional Genes and Soil Properties/Activities

**FIGURE 5:**
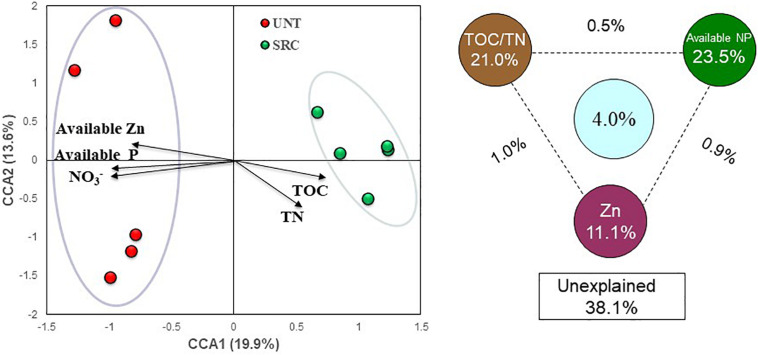
Canonical correspondence analysis (CCA) profile between selected soil parameters and the structure of detected functional genes in the microbial communities of the Unt and SRC soils. CCA-based variation partitioning analysis (VPA) showed the proportions of community structure variations that can be explained by soil organic C, available N and P, and trace element levels of Pb and Zn. The circles show the variation explained by each group of environmental factors alone. The numbers between the circles show the interactions of the two factors on either side.

Concerning linkages between functional gene compositions and soil properties, the CCA (Figure 5) and RDA (Supplementary Figure S4) analyses were performed and consistent results were obtained. In CCA profile, SRC samples clustered separately from Unt samples along the first canonical axis (CCA 1), explaining 19.9% of the total variation in functional gene composition (Figure 5). Projections of soil properties by CCA (*F* = 1.30, *P* = 0.008) showed that microbial functional compositions in SRC were positively correlated with contents of TOC and TN, but negatively correlated with soil NO_3_^–^, available P, and available Zn (Figure 5). Variation partitioning analysis (VPA) based on the CCA revealed that soil TOC and TN explained 21.0% of the total variation in functional gene compositions, while NO_3_^–^, available P and Zn concentrations explained 23.5 and 11.1%, respectively, leaving 38.1% of the total variation in functional gene compositions unexplained.

The Mantel test showed that the composition of all detected functional genes was significantly correlated with NO_3_^–^, available P concentrations, microbial biomass and urease activity (Supplementary Table S1). Concerning groups of functional genes, the composition of C degradation genes was significantly correlated with concentrations of NO_3_^–^, available P, microbial biomass and urease activity. The composition of N cycle genes was significantly correlated with concentrations of NO_3_^–^, available P, microbial biomass, and exchangeable Cr and Cd contents. The composition of genes involved in P utilization was significantly correlated with phosphodiesterase and urease activities, and concentrations of NO_3_^–^ and available P. Significant correlations were also found between the composition of Cd and Zn resistance genes and exchangeable concentrations of Cd and Zn (Supplementary Table S2).

## Discussion

Long-term SRC management with a metal tolerant willow clone led to distinct microbial functional gene compositions, which could be explained by the microbial selection occurring with the fast-growing willow (Berg et al., 2009), as compared to the mixed vegetation of Unt soils. In particular, SRC management resulted in lower HM availability and soil toxicity by BioTox test, likely leading to less metal selective pressure on microbial communities. Correspondingly, lower levels of HM resistance-genes (e.g., Zn resistance genes of *zntA* and *zitB*, Cd, Zn and Co-resistance gene of *czcA*, Cu-resistance genes of *copA*, As-resistance gens of *aoxB, arsA* and *arsC*, Hg-resistance gene of *mer*, Ag-resistance gene of *silC*, Te-resistance gene of *terD*, and Cr-resistance gene of *chrA*) from the microbial communities were observed in SRC than untreated soils. Previously, increased available fractions of HMs were reported to induce positive selection of HM resistance (Nies, 1999; Turpeinen et al., 2004; Azarbad et al., 2016). In this way, we attributed the observed lower abundances of functional genes conferring resistances to As, Cd, Pb, and Zn to microorganisms in SRC soils to their lower HM availability as compared to Unt soils.

Lower abundances of microbial functional genes involved in nutrient cycling were discovered under SRC management, accompanying with lower levels of soil P and N availability. This is possibly a response of soil microbial communities to the intense P and N uptake by the willow plants that depleted soil N and P availability as compared to the native vegetation in Unt. High nutrient uptake efficiency of the Tora willow clone was reported previously (Weih and Dimitriou, 2012), which depleted the available P and N in soils. However, reduced abundances of functional genes involved in nutrient cycling and other genes in soils under phytomanagement as compared to Unt soils were in contrast with those of Epelde et al. (2010) and Xue et al. (2015, 2018) who reported increases in abundances of corresponding microbial functional gene and diversity in HM contaminated soils under phytomanagement. Thus, in HM contaminated soils, phytomanagement effects might be site specific and depend on soil nutrient availability to support the cultivation of HM tolerant plant species. In this case, adequate fertilization could be helpful to improve the efficiency of phytomanagement on the diversity and abundances of functional genes for nutrient cycling in studied SRC soils.

Significantly higher TOC and total N in SRC soils could be explained by the C accumulation potential of the SRC management with woody plants as compared to that of the volunteer vegetation (Lemus and Lal, 2005). This was particularly due to the leaf litter left on the site after stem harvest and willow root proliferation in the topsoil (Rytter and Hansson, 1996), consistent with higher microbial biomass and protease activity. The relatively small response of functional genes involved in C degradation agreed with the observed lack of difference in CO_2_ evolution and C mineralizing enzyme activities between SRC and Unt soils. Moreover, Mantel test results showed that the composition of C degradation genes was significantly correlated with concentrations of NO_3_^–^ and available P, indicating that mineral N and P could be limiting to the microbial decomposition activity (Luo et al., 2006).

According to the German Federal Soil Protection Ordinance (BBodSchV, 1999), the studied soils were contaminated by As, Pb, and Zn and slightly contaminated by Cd, whereas other measured elements presented typical background concentrations of this soil type in Saxony. No significant differences of total HM concentrations between SRC and Unt soils were observed, consistent with findings by Dietzsch (2011), who reported that the willow mediated As, Cd, Pb, and Zn removal from the studied soil after two harvests was negligible. Differently, a general reduction of HM availability was observed in SRC as compared to Unt soils due to both plant absorption and stabilization effects of the plant-derived organic matter (Mench et al., 2010), although their availability still exceeded the Trigger and Action values of the German environmental legislation (Pruess, 1995). In particular, the Zn availability was significantly reduced in SRC soils as compared to Unt soils. This is mainly ascribed to Zn’s higher mobility in soils and ease of absorption and translocation by willow plants (Vysloužilová et al., 2003), as compared to other HMs such as Cr and Pb that are less mobile and less absorbed by plants (Dimitriou et al., 2006).

Lower abundances of microbial functional genes conferring resistances to un-contaminated HMs (e.g., Cu, Hg, Ag, and Cr) in SRC as compared to Unt soils could be explained by the fact that multiple HM resistance genes are often present in the same organism (Mergeay, 2000), or by horizontal gene transfer of HM resistance genes that confers increased HM resistances on their recipients, e.g., through mobilized plasmid (Dong et al., 1998). For example, the abundance of the czcA conferring resistance to Cd, Co, and Zn was significantly lower in SRC soils, and the reduction of organisms resistant to Zn may lead to loss for additional resistance to Co in microbial communities of soils under SRC phytomanagement.

Analogously, the observed reduction of genes encoding for antibiotic resistance in microbial communities of SRC soils as compared to Unt soils could be ascribed to the fact that microbial genes encoding for HM and antibiotic resistance are often located on the same mobile genetic elements such as plasmids (Mergeay, 2000). In addition, the typical HMs (e.g., Cu) were observed to promote the conjugative transfer of environmental-mediated plasmid RP4 via inducing cell damage, thus facilitating the transmission of ARGs (Wang et al., 2020). Thus, we argued that the adopted phytomanagement relieved the HM-induced stress by lowering the HM availability, which in turn reduced the abundance of antibiotic resistance genes due to their genetic linkage to HM resistance genes in microorganism (Hobman and Crossman, 2014) and horizontal gene transfer.

In conclusion, though the SRC management with woody plants did not reduce the total HM concentrations in soils, it reduced their availability and soil toxicity as compared to the same soil under mixed vegetation representing the no intervention scenario. Thus, the SRC phytomanagement reduced abundances of HM resistance genes in soil microbial communities, indicating a lower selective pressure on the microbial communities as compared to Unt soils. The N and P depletion caused by the uptake by fast growing willow plants reduced the abundance of genes involved in nutrient cycling, and appeared to limit microbial carbon decomposition. Therefore, the recovery of microbial functional gene diversity in soils under phytomanagement may be site specific and require soil stewardship.

## Data Availability Statement

All datasets generated for this study are included in the article/Supplementary Material. The GeoChip dataset is available in: http://129.15.40.240/NewIEGWebsiteFiles/publications/SupplData/KaiXue2020GermanGeoChip.csv.

## Author Contributions

All authors contributed intellectual input and assistance to this study and manuscript preparation. JV and GR developed the original concepts. KX, JV, SN, IM, and LG contributed reagents, experimental conduction, experimental test, data collection, and data analysis. Specifically, SN, IM, and LG handled all soils processing and subsampling for soil properties tests and microbial eco-toxicological test. KX and JV handled soil subsamples for microbial functional gene analysis. KX and GR performed data analysis and wrote the manuscript with help from JZ and LG. All authors were involved in revising this manuscript. All authors contributed to the article and approved the submitted version.

## Conflict of Interest

The authors declare that the research was conducted in the absence of any commercial or financial relationships that could be construed as a potential conflict of interest.
