# Supplementary material for: Phytomanagement Reduces Metal Availability and Microbial Metal Resistance in a Metal Contaminated Soil
Source: Front Microbiol. 2020 Aug 7;11:1899. doi: 10.3389/fmicb.2020.01899 (PMC7426507; doi:10.3389/fmicb.2020.01899)
Supplement: Supplementary file 1 [file Data_Sheet_1.docx]

**Phytomanagement reduces metal availability and microbial metal resistance in a metal contaminated soil**

Kai Xue^1,2,3^, Joy D. Van Nostrand^4^, Jizhong Zhou^4^, Silke Neu^5^, Ingo Müller^5^, Laura Giagnoni^6^, Giancarlo Renella^7*^

^1^ Key Laboratory of Environmental Biotechnology, Chinese Academy of Sciences (CAS), Beijing, China

^2^CAS Center for Excellence in Tibetan Plateau Earth Sciences, Chinese Academy of Sciences (CAS), Beijing, China

^3^College of Resources and Environment, University of Chinese Academy of Sciences, Beijing, China

^4^Institute for Environmental Genomics and Department of Botany and Microbiology, University of Oklahoma, Norman, OK, USA

^5^Saxon State Office for Environment, Agriculture and Geology, Freiberg, Germany

^6^Department of Agrifood Production and Environmental Sciences, University of Florence, Florence, Italy

^7^Department of Agronomy, Food, Natural Resources, Animals and Environment (DAFNAE), Agripolis Campus, University of Padova, Legnaro, Italy

*Correspondence:

Dr. Giancarlo Renella

[Giancarlo.renella@unipd.it](mailto:Giancarlo.renella@unipd.it);

**Supplementary Text**

1. **Materials and methods**
   1. **Analysis of soil chemical properties**

Pseudototal HMs were extracted by soil microwave assisted digestion (Ethos, Milestone) with the USEPA 3051 method, whereas the HM availability was determined by extractions with 1M NH_4_NO_3_ (ISO19730 2008; Pruess 1995), buffered at pH 7.00 with concentrated ammonia (Renella et al., 2004). The HM concentrations were quantified by ICP optical spectrometer (Vista MPX, Varian). The total organic C (TOC) was determined by the method of Walkley and Black (Walkley and Black 1934), whereas the total N was determined using a CHN-S Flash E1112 elemental analyzer (Thermo Finnigan) with a standard method (ISO10694, 1995). Inorganic N (NH_4_^+^ and NO_3_^-^) was extracted by shaking 5 g soil for 1 h with 1M KCl (1:5 soil:solution ratio) according to Keeney and Nelson (Keeney and Nelson 1982), and quantified by using ion selective electrodes (Crison, Spain). Total soil P concentration was determined by the H_2_O_2_/H_2_SO_4_ - H_2_SO_4_/HF wet digestion method (Browman and Tabatabai, 1978), and phytoavailable P concentration was estimated by HCl/NH_4_F extractions (Bray and Kurtz 1945). Both total and available P were quantified colorimetrically at 880 nm after reaction of the extracts with the sulfo-molybdic acid reagent (Murphy and Riley 1962).

**1.2 Analysis of soil microbial biomass and enzyme activities**

Soil respiration rate was estimated by the CO_2_ evolution determined by gas chromatography (HP 5890) according to (Blackmer and Bremner 1977). Soil microbial biomass was estimated by determining the adenosine triphosphate (ATP) content according to Ciardi and Nannipieri (1990) using the same sample previously used for measuring the soil respiration. The arylesterase activity was determined as described by Zornoza et al. (2009), the acid and alkaline phosphomonoesterase activities were assayed according to Tabatabai and Bremner (1969), the phosphodiesterase activity as reported by Browman and Tabatabai (1978). The β-glucosidase and β-galactosidase activities were assayed according to Tabatabai (1982). Urease and protease activities were determined according to Nannipieri et al. (1974) and Ladd and Butler (1972), respectively.

**1.3 Analysis of functional gene abundance and diversity**

Abundance and diversity of functional genes of soil microbial communities were analyzed by GeoChip 4.2 on DNA extracted by freeze-grinding mechanical lysis (Zhou et al., 1996). The chip contained 107.950 probes, covering 792 functional gene families from 11 major functional categories, including C, N, P and S cycling categories, plus HMs and antibiotic resistance genes (Tu et al. 2014). Full details on the DNA labeling, hybridization, image analysis and data processing were reported previously (Xue et al. 2015, 2018).

**1.4 Data analysis**

The soil chemical and biochemical data were analyzed by ANOVA followed by the Fisher LSD HMst for sample comparisons. The functional gene diversity of the microbial communities in Unt and SRC soils was assessed by calculating various ecological indices such as richness (detected probe number), Shannon–Weaver (H), Simpson Reciprocal (1/D) and Eveness (1/H) indices. For the calculation of diversity indexes, the detected gene probes were considered as species and their abundances were represented by the normalized signal intensities. Changes in functional gene composition under Unt and SRC management were assessed by the Detrended Correspondence Analysis (DCA) and the non-parametric similarity tests multiple response permutation procedure (MRPP), permutational multivariate analysis of variance (Adonis) and analysis of similarity (ANOSIM) based on Bray-Curtis, Horn and Euclidean dissimilarity indices. Analysis of variance (ANOVA) was used to compare the normalized functional gene abundances, where individual probes were included as a factor to partition the variance from various probes within each gene catalogue. The linkage between microbial functional gene composition and soil properties was assessed by the canonical correlation analysis (CCA) and Mantel test. All statistics and modeling were performed using R version 3.0.2 (The R Foundation for Statistical Computing, Vienna, Austria), and significant differences were defined as *P* < 0.05.

1. **Functional gene abundance and diversity**

Most of genes (29 of 33) encoding enzymes for degrading organic C compounds did not significantly differ in their abundance between soils under Unt or SRC management, whereas genes encoding for cyclomaltodextrinase (*cda*) involved in starch degradation, endoglucanase involved in cellulose degradation, isocitrate lyase (*aceA*) involved in aromatic compound degradation, and glyoxal oxidase (*glx*) involved in lignin degradation, showed significantly lower abundances in SRC than in Unt soils (Fig. S1).

About functional genes involved in N cycling, those encoding the nitrite reductase (*nirA*) for assimilatory N reduction, the nitrate reductase subunit alpha (*narG*), the nitrite reductase (*nirS* and *nirK*) involved in denitrification, bacterial ammonia monooxygenase (*amoA*), hydroxylamine oxidoreductase (*hao*) involved in nitrification, and nitrogenase (*nifH*) involved in N_2_ fixation were significantly lower in the SRC than Unt soils (Fig. S2).

Among the functional genes involved in P cycling, the gene encoding exopolyphosphatase (*ppx*) for phosphorus utilization was significantly less abundant (*P* ≤ 0.05) in SRC than Unt soils (Fig. S3). Concerning the functional genes involved in S cycling, *cysI, dsrB* and *dsrA* encoding the sulfite reductase showed significantly lower abundance in SRC than Unt soils; differently, the *cysJ* encoding the sulfite reductase was significantly more abundant in SRC than Unt soils (Fig. S3).

Blackmer A.M., Bremner J.M. (1977) Gas chromatographic analysis of soil atmosphere. Soil Sci. Soc. Am. J. 41: 908-912.

Bray R.H., Kurz L.T. (1945) Determination of total organic and available forms of phosphorus in soils. Soil Sci. 59: 39-45.

Browman M.G., Tabatabai M.A. (1978) Phosphodiesterase activity of soils. Soil Sci. Soc. Am. J. 42: 284-290.

Ciardi C., Nannipieri P. (1990) A comparison of methods for measuring ATP in soil. Soil Biol. Biochem. 22:725-727.

Keeney D.R., Nelson D.W.(1982) Nitrogen-inorganic forms. In *Agron Monogr, 2nd ed, vol 9*; Page, A.L., Miller, R.H., Keeney, D.R., Eds.; ASA and SSSA: Madison, WI, USA. pp 643-698.

Ladd J.N., Butler J.H.A. (1972) Short-term assays of soil proteolytic enzyme activities using proteins and dipeptide derivatives as substraHMs. Soil Biol. Biochem. 4:19-30.

Lappalainen J., Juvonen R., Vaajasaari K., Karp M. (1999) A new flash method for measuring the toxicity of solid and colored samples. Chemosphere 38:1069-1083.

Murphy J., Riley J.P. (1962) A modified single solution method for the determination of phosphate in natural waters. Anal. Chim. Acta. 27: 31-36.

Nannipieri P., Ceccanti B., Cervelli S., Sequi P. (1974) Use of 0.1 M pyrophosphate to extract urease from a podzol. Soil Biol. Biochem. 6: 359-362.

Quershi A.A., Bulich A.A., Isenberg D.L. (1998) Microtox toxicity test systems— where they stand today. In: Wells PG, Lee K, Blaise C, editors. Microscale testing in aquatic toxicology advances, techniques, and practice. Boca Raton: CRC Press, pp 185-199.

Renella G., Adamo P., Bianco M.R., Landi L., Violante P., Nannipieri P. (2004) Availability and speciation of cadmium added to a calcareous soil under various managements. Eur. J Soil Sci. 55:123-133.

Tabatabai M.A., Bremner J.M. (1969) Use of p-nitrophenyl phosphate for assay of soil phosphatase activity. Soil Biol. Biochem. 1: 301-307.

Tabatabai M.A. (1982) Soil enzymes. In *Chemical and Microbiological Properties, 2nd Edition* *Methods of Soil Analysis. Part 2*; Page A.L., Miller R.H., Keeney D.R., Eds.; American Society of Agronomy/Soil Science Society of America: Madison, WI 1982, pp 903-947.

Tu Q., Yu H., He Z., Deng Y., Wu L., van Nostrand J.D., Zhou A., Voordeckers J., Lee Y.J., Qin Y., Hemme C.L., Shi Z., Xue K., Yuan T., Wang A., Zhou J.Z. (2014) GeoChip 4: a functional gene-array-based high-throughput environmental technology for microbial community analysis. Mol. Ecol. Res. 14: 914–928; DOI: 10.1111/1755-0998.12239.

Walkley A., Black I.A. (1934) An examination of the Degtjareff method for determining organic carbon in soils: Effect of variations in digestion conditions and of inorganic soil constituents. Soil Sci. 63: 251-263.

Zhou J.Z., Bruns M.A., Tiedje J.M. (1996). DNA recovery from soils of diverse composition. Appl Environ Microbiol. 62:316-322.

Zornoza R., Landi L., Nannipieri P., Renella G. (2009) A protocol for the assay of arylesterase activity in soil. Soil Biol. Biochem. 41: 659-662.

**Supplementary figures**

**Figure S1.** Normalized signal intensity of detected functional genes encoding enzymes involved in carbon substrate degradation in Unt and SRC soils. The complexity of C substrates is presented in order from labile to recalcitrant from left to right. Error bars represent standard error. Symbols * indicates significant differences at P < 0.05.

**
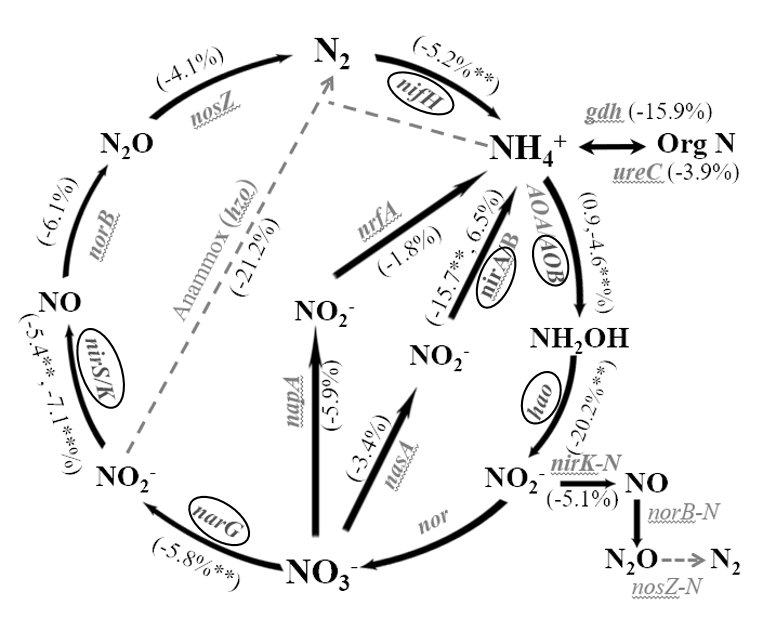
**

**Figure S2.** Percentage change of normalized signal intensity from detected functional genes involved in nitrogen cycling in Unt and SRC soils. The genes in circled font indicate significant Unt > SRC and Symbols ** indicates significant differences at *P* < 0.05.

**Figure S3.** Normalized signal intensity of detected functional genes encoding enzymes involved in sulphur and phosphorus cycles in Unt and SRC soils. Error bars represent standard error. Symbols ** indicates significant differences at P < 0.05. The circled ** symbols indicate significant differences at P < 0.05, with red color when SRC < Unt, with blue color when SRC > Unt.


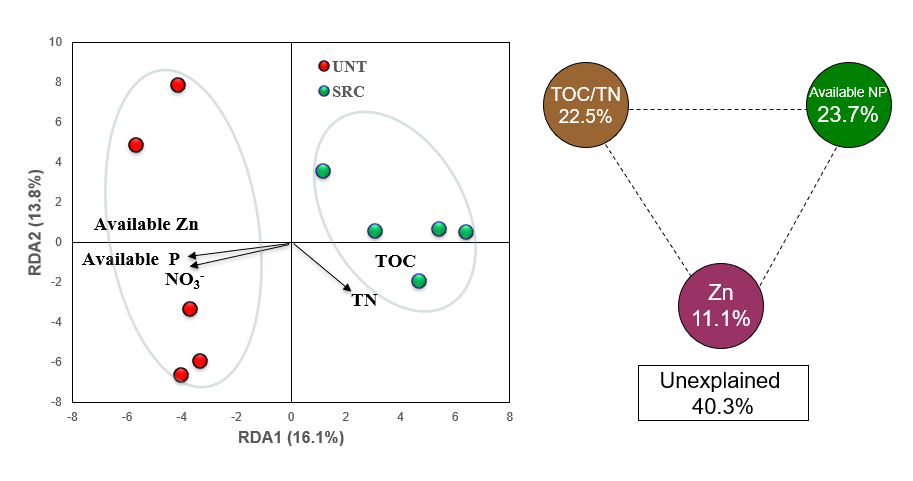


**Figure S4.** Redundancy analysis (RDA) profile between selected soil parameters and the structure of detected functional genes in the microbial communities of the Unt and SRC soils. RDA-based variation partitioning analysis (VPA) showed the proportions of community structure variations that can be explained by soil organic C, available N and P, and trace element levels of Pb and Zn. The circles show the variation explained by each group of environmental factors alone. The numbers between the circles show the interactions of the two factors on either side.

Table S1. Correlation between the composition of certain functional gene groups (Carbon degradation, Nitrogen, Phosphorus and Sulphur cycling genes) and enzyme activities or soil properties by Mantel test. Symbols * indicates significant correlations at P < 0.05.

|  | **C degradation** | **Nitrogen** | | | | | | | | **Phosphorus** | **Sulphur** |
| --- | --- | --- | --- | --- | --- | --- | --- | --- | --- | --- | --- |
|  |  | **whole** | Assimilatory N reduction | Denitrification | Dissimilatory N reduction | N fixation | Nitrification | Ammonification | Anammox |  |  |
| Arylesterase |  |  |  |  |  |  |  |  |  |  |  |
| Arylsulfatase |  |  |  |  |  |  |  |  |  |  |  |
| Acid phosphatase |  |  |  |  |  |  |  |  |  |  |  |
| Alkaline phosphatase |  |  | * |  | * |  |  |  |  |  |  |
| phosphodiesterase |  |  |  |  |  |  |  |  |  | ***** |  |
| b-glucosidase |  |  | * |  |  |  |  |  |  |  |  |
| b-galactosidase |  |  |  |  |  |  |  |  |  |  |  |
| urease | ***** |  |  | * |  |  |  | * |  | ***** | ***** |
| protease |  |  |  |  |  |  |  |  |  |  |  |
| ATP | ***** | ***** | * | * | * |  | * |  |  |  | ***** |
| CO_2_ |  |  |  |  |  |  |  |  |  |  |  |
| BioTox (%) |  |  |  |  |  |  |  |  |  |  |  |
| organic C |  |  |  |  | * |  |  |  |  |  |  |
| total N |  |  |  |  |  |  |  |  |  |  |  |
| NH_4_- |  |  | * |  |  |  |  |  |  |  |  |
| NO_3_- | ***** | ***** | * | * | * | * | * | * | * | ***** | ***** |
| total P |  |  |  |  |  |  |  |  |  |  |  |
| Olsen P | ***** | ***** | * | * | * | * | * | * | * | ***** | ***** |
| soluble P |  |  | * |  |  |  |  |  |  |  |  |
| organic P |  |  | * |  |  |  |  |  |  |  |  |
| As_tot |  |  |  |  |  |  |  |  |  |  |  |
| Cd_tot |  |  |  |  |  |  |  |  |  |  |  |
| Cr_tot |  |  |  |  |  |  |  |  |  |  |  |
| Cu_tot |  |  |  |  |  |  |  |  |  |  |  |
| Mn_tot |  |  |  |  |  |  |  |  |  |  |  |
| Ni_tot |  |  |  |  |  |  |  |  |  |  |  |
| Pb_tot |  |  |  |  |  |  |  |  |  |  |  |
| Se_tot |  |  |  |  |  |  |  |  |  |  |  |
| Zn_tot |  |  |  |  |  |  |  |  |  |  |  |
| As_sol |  |  |  |  |  |  |  |  |  |  |  |
| Cd_sol |  |  |  |  |  |  |  |  |  |  |  |
| Cr_sol |  | ***** |  |  | * | * | * |  |  |  |  |
| Ni_sol |  |  |  |  |  |  |  |  |  |  |  |
| Pb_sol |  |  |  |  |  |  |  |  |  |  |  |
| Zn_sol |  |  |  |  | * | * |  |  |  |  |  |
| As_exchang |  |  |  |  |  |  |  |  |  |  |  |
| Cd_exchang |  | ***** | * |  | * |  |  |  | * |  |  |
| Cr_exchang |  |  |  |  |  |  |  |  |  |  |  |
| Ni_exchang |  |  |  |  |  |  |  |  |  |  |  |
| Pb_exchang |  |  |  |  |  |  |  |  |  |  |  |
| Zn_exchang |  |  |  |  |  |  |  |  |  |  |  |

Table S2 Correlation between the composition of certain functional gene groups (Metal resistance and antibiotic resistance genes) and enzyme activities and soil properties by Mantel test. Symbols * indicates significant correlations at P < 0.05.

|  | **Metal Resistance** | | | | | | **Antibiotic resistance** |
| --- | --- | --- | --- | --- | --- | --- | --- |
|  | **Whole** | Arsenic | Cadmium | Cadmium,Cobalt,Zinc | Zinc | Lead |  |
| Arylesterase |  |  |  |  |  |  |  |
| Arylsulfatase |  |  |  |  |  |  |  |
| Acid phosphatase |  |  |  |  |  |  |  |
| Alkaline phosphatase |  |  |  |  | * |  |  |
| phosphodiesterase |  |  |  |  |  |  |  |
| b-glucosidase |  |  |  |  |  |  |  |
| b-galactosidase |  |  |  |  |  |  |  |
| urease | ***** |  | * |  | * | * |  |
| protease |  |  |  |  |  |  |  |
| ATP | ***** | * | * |  | * |  |  |
| CO_2_ |  |  |  |  |  |  |  |
| BioTox (%) |  | * |  |  |  |  |  |
| organic C |  |  |  | * | * |  |  |
| total N |  |  |  |  |  |  |  |
| NH_4_- |  |  |  |  | * |  |  |
| NO_3_- | ***** |  | * | * | * |  | ***** |
| total P |  |  |  |  |  |  |  |
| Olsen P | ***** |  | * | * | * |  | ***** |
| soluble P |  |  |  |  |  |  |  |
| organic P |  | * |  |  | * |  |  |
| As_tot |  |  |  |  |  |  |  |
| Cd_tot |  |  |  |  |  |  |  |
| Cr_tot |  |  |  |  |  | * |  |
| Cu_tot |  |  |  |  |  |  |  |
| Mn_tot |  |  |  |  |  |  |  |
| Ni_tot |  |  |  |  |  |  |  |
| Pb_tot |  |  |  |  |  |  |  |
| Se_tot |  |  |  |  |  |  |  |
| Zn_tot |  |  |  |  |  |  |  |
| As_sol |  |  |  |  |  |  |  |
| Cd_sol |  | * |  |  |  |  |  |
| Cr_sol |  |  |  | * |  |  | ***** |
| Ni_sol |  |  |  |  |  |  |  |
| Pb_sol |  |  |  |  |  |  |  |
| Zn_sol |  |  |  |  | * |  |  |
| As_exchange |  |  |  |  | * |  |  |
| Cd_exchange |  |  | * |  | * |  |  |
| Cr_exchange |  |  |  |  |  |  |  |
| Ni_exchange |  |  |  |  | * |  |  |
| Pb_exchange |  |  |  |  | * |  |  |
| Zn_exchange |  |  |  |  | * |  |  |

Table S3 Micorbial fucnional genes signficantly correlatd with DCA1 in catalogs of Carbon cycling, Nitrogen, Antibiotic resistane, Metal Resistance and Stress.

| **Catalog** | **Sub-Catalog1** | **Sub-Catalog2** | **Gene** | **r** | **p** |
| --- | --- | --- | --- | --- | --- |
| Antibiotic resistance | Beta-lactamases |  | B_lactamase_A | -0.708 | 0.022 |
| Carbon cycling | Carbon degradation | Cellulose | exoglucanase | -0.658 | 0.039 |
|  | Carbon degradation | Lignin | *glx* | -0.830 | 0.003 |
|  | Carbon degradation | Starch | *cda* | -0.719 | 0.019 |
|  | Carbon degradation | Starch | *nplT* | -0.655 | 0.040 |
|  | Carbon fixation |  | *aclB* | -0.730 | 0.016 |
|  | Carbon fixation |  | CODH | -0.795 | 0.006 |
| Metal Resistance | Aluminum |  | Al | -0.707 | 0.022 |
|  | Arsenic |  | *aoxB* | -0.815 | 0.004 |
|  | Arsenic |  | *ArsA* | -0.841 | 0.002 |
|  | Arsenic |  | *arsB* | -0.682 | 0.030 |
|  | Arsenic |  | *arsM* | 0.734 | 0.016 |
|  | Cadmium,Cobalt,Zinc |  | *czcA* | -0.703 | 0.023 |
|  | Chromium |  | *ChrA* | -0.650 | 0.042 |
|  | Copper |  | *CusA* | 0.681 | 0.030 |
|  | Mercury |  | *mer* | -0.711 | 0.021 |
|  | Silver |  | *silC* | -0.678 | 0.031 |
|  | Zinc |  | *ZitB* | -0.861 | 0.001 |
|  |  |  | *SilE* | -0.831 | 0.003 |
| Nitrogen | Ammonification |  | *gdh* | -0.715 | 0.020 |
|  | Anammox |  | *hzo* | -0.672 | 0.033 |
|  | Assimilatory N reduction |  | *nirA* | -0.819 | 0.004 |
|  | Nitrification |  | *hao* | -0.861 | 0.001 |
| Stress | Nitrogen limitation |  | *glnR* | -0.855 | 0.002 |
|  | Oxygen limitation |  | *cydA* | -0.785 | 0.007 |
|  | Oxygen stress |  | *katE* | -0.636 | 0.048 |
|  | Oxygen stress |  | *oxyR* | -0.726 | 0.017 |
|  | Phosphate limitation |  | *phoB* | -0.659 | 0.038 |
|  | Sigma factors |  | sigma_32 | -0.806 | 0.005 |
